# Suppression of Cornea Stromal Fibrosis by Vitamin D

**DOI:** 10.3390/cells14201583

**Published:** 2025-10-11

**Authors:** Xiaowen Lu, Zhong Chen, Jerry Lu, Mitchell A. Watsky

**Affiliations:** Department of Cellular Biology and Anatomy, Medical College of Georgia, Augusta University, Augusta, GA 30912, USA; xialu@augusta.edu (X.L.); zchen@augusta.edu (Z.C.); zl958@cornell.edu (J.L.)

**Keywords:** vitamin D, cornea, wound healing, keratocyte, myofibroblast, fibrosis, bone morphogenic protein, TGF

## Abstract

Corneal fibrosis, a significant source of visual impairment, can result from keratocyte-to-myofibroblast transdifferentiation during wound healing. This study investigated the antifibrotic role of 1,25-dihydroxyvitamin D_3_ (1,25 Vit D) and the lesser-known vitamin D, 24,25-dihydroxyvitamin D_3_ (24,25 Vit D), in human and mouse corneal stromal cells (HSCs and MSCs) and in a Vit D receptor knockout (VDR KO) mouse model. Cells were treated with TGF-β1 ± Vit D metabolites and the expression of fibrotic and antifibrotic genes and proteins was evaluated. Both metabolites significantly reduced α-smooth muscle actin levels in HSCs, MSCs and organ-cultured mouse corneas (*p* < 0.05). They also upregulated the mRNA expression of BMP2, BMP6, BMPR2, and TGF-β3, as well as the protein expression of BMP6 and TGF-β3. VDR KO corneas subjected to alkali injury exhibited increased fibrotic responses and reduced CD45+ immune cell infiltration compared to wild-type controls. Notably, 24,25 Vit D exerted antifibrotic effects even in VDR KO cells, and the alternative 24,25 Vit D receptor FAM57B was expressed in all corneal cell layers. These results reveal consistent antifibrotic effects of both 1,25 and 24,25 Vit D across species, support the existence of VDR-independent mechanisms in the cornea, and offer new insights into potential therapeutic strategies for preventing corneal fibrosis.

## 1. Introduction

Corneal opacity, which can occur following trauma, chemical injury, infection, or surgery, is a leading cause of vision loss; thus, enhancing treatments that promote the resolution of stromal fibrosis-induced corneal scarring is crucial [1]. The pathophysiologic process of corneal fibrosis/scarring involves differentiation of corneal keratocytes into fibroblasts and myofibroblasts, which leads to the deposition of disorganized collagen fibrils and extracellular matrix (ECM) [2]. Vitamin D (Vit D), a multifunctional hormone that plays an essential role in calcium homeostasis, immune system regulation and cell differentiation, has been shown to modulate liver fibrosis, lung fibrosis, and cutaneous scar formation [3,4,5,6,7,8]. These modulating roles in fibrosis suggest that Vit D may be able to reduce stromal fibrosis-induced scarring and resolve corneal haze, alluding to future potential therapeutic applications in ocular surgeries.

The keratocyte-to-myofibroblast transition plays a central role in corneal fibrosis [9,10,11]. Antifibrotic factors, which maintain corneal transparency by regulating fibrotic events, are essential for corneal wound healing. By reducing inflammation and promoting ordered stromal regeneration, these factors support proper repair and restore corneal clarity after injury or surgery. TGF-β superfamily bone morphogenetic proteins, including BMP2, BMP6 and TGF-β3, are established antifibrotic factors in the cornea, effectively reducing collagen deposition, strongly modulating fibroblast activity, and potently suppressing myofibroblast differentiation [12,13]. BMP signaling occurs primarily through bone morphogenetic protein receptor type 2 (BMPR2), a critical receptor that enhances cellular responsiveness to BMPs [12]. Signaling events at BMPR2 robustly inhibit fibrosis by counteracting pro-fibrotic TGF-β-driven responses and promoting antifibrotic gene expression. Thus, BMPR2-mediated BMP signaling is highly effective in suppressing collagen deposition, regulating fibroblast behavior, and potently inhibiting myofibroblast differentiation.

Many tissues in the eye can both activate and respond to Vit D [14] and Vit D deficiency has been associated with a wide range of ocular pathologies, including myopia [15], age-related macular degeneration [16,17], diabetic retinopathy [18], uveitis [19], and glaucoma [20]. Previous studies have shown that Vit D may play a protective role in ocular health [21]. These roles include inhibition of angiogenesis in transgenic murine retinoblastoma [22], inhibition of corneal neovascularization induced by sutures [23] and retinal neovascularization in murine eyes [24], prevention of retinal autoimmune disease and mitigation of uveitis [25], and decreased retinal inflammation and levels of amyloid beta accumulation [26], as well as lowering intraocular pressure in primates [27].

1,25-dihydroxyvitamin D3 (1,25 Vit D), the long recognized active Vit D metabolite, has been found to induce keratinocyte differentiation and proliferation in skin [28]. It also regulates ECM turnover and mediates matrix metalloproteinase (MMP) production, including MMP-2 and MMP-3, during cell maturation and hypertrophy [29]. 1,25 Vit D was also found to significantly reduce the expression levels of α-smooth muscle actin (α-SMA) and fibronectin in a dose-dependent manner in TGF β1-induced fibroblasts derived from nasal polyps [30]. In the Vit D metabolic pathway, both 25-hydroxyvitamin D3 and 1,25 Vit D can be metabolized by 24-hydoxylase to form 24,25-dihydroxyvitamin D3 (24,25 Vit D). It has only recently been recognized that 24,25 Vit D is more than a metabolic byproduct of Vit D metabolism, as 24,25 Vit D injections have been shown to restore callus volume, stiffness, and mineralized cartilage area in cytochrome P450 family 24 subfamily A member 1 null mice [31]. Our group previously identified that 24,25 Vit D is a prevalent Vit D metabolite in the rabbit eye and demonstrated that 24,25 Vit D enhances corneal epithelial cell proliferation and enhances gap junction communication [32,33]. We also described significant effects of 1,25 Vit D and 24,25 Vit D in VDR KO corneal epithelial cells, and attributed those findings to the presence of alternative Vit D receptors in these cells [32,33]. The current study examines the hypothesis that 1,25 Vit D and 24,25 Vit D can alter the progression of fibrosis following wound healing. To the best of our knowledge, this is the first study to examine the possible antifibrotic effects of 24,25 Vit D. Along the same line, this study also examines alternative Vit D receptors in keratocytes, which also have not been studied regarding their anti-fibrotic properties.

FAM57B has recently emerged as a putative receptor for 24,25 Vit D, providing new insight into noncanonical Vit D signaling pathways. Specifically, studies revealed that 24,25 Vit D binds to FAM57B_2_, triggering lactosylceramide production, which is essential for cartilage maturation and callus formation [31]. In the context of corneal injury, where VDR expression may be altered or absent, FAM57B-mediated signaling could represent a compensatory or complementary pathway by which 24,25 Vit D exerts protective, antifibrotic effects. Elucidating the functional role of FAM57B in this setting could significantly expand our understanding of vitamin D biology and its therapeutic potential in ocular and fibrotic diseases.

Resident immune cells in the corneal epithelium and stroma include dendritic cells, macrophages, mast cells, and innate lymphoid cells [34,35]. Following corneal epithelial debridement in mice, large numbers of CD45+ cells infiltrate the stroma, including monocytes, macrophages, neutrophils, lymphocytes, and stem cells [36,37,38]. These infiltrating CD45+ cells play critical roles in the wound healing process by modulating inflammation, clearing cellular debris and pathogens, secreting cytokines and growth factors, and influencing keratocyte activation and differentiation [39]. A recent study from our group found that CD45+ cells with dendriform morphology were significantly reduced in the basal epithelial layer of wounded diabetic mouse corneas compared to normoglycemic mice, with topical 24,25 Vit D increasing their numbers [40].

Although Vit D metabolites (1,25 Vit D and 24,25 Vit D) demonstrate antifibrotic effects in extraocular tissues through VDR-SMAD3 inhibition and BMP-SMAD1/5 activation [41,42,43], their role in corneal TGF-β/BMP signaling remains uncharacterized. Crucially, FAM57B, a receptor for 24,25 Vit D, is detectable in osteoblasts and chondrocytes [31] but unstudied in corneal cells. This study tests the hypothesis that 1,25 Vit D and 24,25 Vit D attenuate corneal myofibroblast differentiation via VDR-dependent and FAM57B-mediated pathways, respectively, by modulating BMP/TGF-β3 expression and immune cell recruitment. We explored the effects of Vit D on the corneal stroma, specifically examining the role of Vit D metabolites in corneal keratocyte to myofibroblast transition and corneal fibrosis after wounding.

## 2. Materials and Methods

### 2.1. Experimental Design

This study used a multi-method approach to test the hypothesis that Vit D can influence corneal wound healing and fibrosis, as well as the transition of keratocyte to myofibroblast. Cell culture studies were performed in a controlled environment to compare results between human and mouse stromal cells. Organ culture was utilized to move those studies from cells to whole corneas. In vivo studies were performed to determine if VDR KO influences immune cell infiltration and fibrotic wound healing in the live animal, an environment impossible to mimic in vitro.

### 2.2. Materials

The following reagents were procured from their respective suppliers: 1,25 Vit D and 24,25 Vit D were sourced from Enzo Life Sciences (#BML-DM200-0050, BML-DM300-0050, Farmingdale, NY, USA). Primary antibodies included anti-α-SMA monoclonal antibody, anti-CD45 polyclonal antibody, and anti-BMP6 monoclonal antibody (ABCAM, #ab32575, ab10558, and ab155963, Waltham, MA, USA), anti-type III Collagen-UNLB (SouthernBiotech, #1330-01, Birmingham, AL, USA), anti-fibronectin (Millipore sigma, #6140, St. Louis, MO, USA), anti-FAM57B2 (used in the recent J. Clin. Invest. study to identify this protein as a 24,25 Vit D receptor [31]; Biorbyt, #orb183613, Durham, NC, USA), and anti-TGF β3 polyclonal antibody (ThermoFisher Scientific, #PA5-32630, Waltham, MA, USA). Secondary antibodies included goat anti-rabbit IgG (H + L) Alexa Fluor 488, donkey anti-Goat IgG (H + L) Alexa Fluor™ 555, goat anti-Mouse IgG (H + L) Alexa Fluor™ 633, and peroxidase-conjugated anti-rabbit IgG secondary antibody (ThermoFisher Scientific, #A-11034, #A-21432, #A-21052, #31460).

### 2.3. Animal Experiments

VDR knockout (VDR KO) mice were obtained from The Jackson Laboratory (B6.129S4-Vdrtm1Mbd/J Strain #: 006133). Wild type (WT) littermates were used as controls for all studies. All animal procedures were approved by the Augusta University Institutional Animal Care and Use Committee (protocol number: 2013-0581) and adhered to the ARVO Statement for the Use of Animals in Ophthalmic and Visual Research (https://integration.arvo.org/About/policies/statement-for-the-use-of-animals-in-ophthalmic-and-vision-research/ accessed on 21 April 2025). The animals were housed under standard conditions with a 12-h dark-light cycle. At the conclusion of the experiments, mice were euthanized by CO_2_ inhalation followed by cervical dislocation.

### 2.4. Cornea Fibroblast Cell Culture

Primary human and mouse cornea stromal cells and a human cornea stroma cell line were used for this study. Primary human stromal cells were obtained from multiple donors (males ages 32 yr, 33 yr, 58 yr). The stromal cell line has been previously described [44,45], and the isolation and culturing methods for the human and mouse primary cornea stromal fibroblasts (HSCs and MSCs) have been described in detail [46]. Briefly, donor human cornea rims and mouse corneas from four 4-week-old WT and VDR KO C57BL/6 mice were minced with scissors and placed in DMEM (ThermoFisher Scientific, #11965118) plus 1% fetal bovine serum (ThermoFisher Scientific, #SH3007103HI), and incubated at 37 °C with 5% CO_2_, allowing for migration of the cells out of the tissue and onto an uncoated culture dish. To confirm the keratocyte lineage, cells were routinely screened using western blotting for the corneal stromal cell-specific marker keratocan and the fibroblast marker Thy1/CD90 [47,48]. Primary mouse cornea stromal cells were from 6 mice. For all cell culture experiments, cell passages 2–6 were used.

Use of de-identified human donor cornea rims was deemed exempt by the Augusta University IRB committee. All protocols for animal use and euthanasia were reviewed and approved by the Augusta University Institutional Animal Care and Use Committee and followed the ARVO Statement for the Use of Animals in Ophthalmic and Vision Research.

### 2.5. Corneal Fibroblast Vit D Treatment

MSCs and human stroma cell line HSCs were treated with 1,25 Vit D (20 nM in DMSO) or 24,25 Vit D (100 nM in DMSO) and 10 ng/mL TGF β1 for 24 h (n = 5). We have found these concentrations and the 24-h time point to be effective in previous studies [49]. DMSO was administered to the control groups as the solvent control.

### 2.6. Antifibrotic Factor Expression in Primary HSCs Treated with Vit D

To investigate the effects of Vit D on antifibrotic factors (BMP2, BMP6, BMPR2, and TGFβ3), HSCs were treated with either 20 nM 1,25 Vit D or 100 nM 24,25 Vit D along with 10 ng/mL TGF β. RNA was extracted from the cells using TRIzol (ThermoFisher Scientific, #15596026) at 2-, 6-, and 12-h post-treatment to assess gene expression. Q-PCR was performed to analyze gene expression levels using three biological replicates for each condition. Gene expression levels in the treated group were normalized to those in the control group. Each sample was analyzed using technical triplicates to ensure accuracy and reproducibility. For antifibrotic factor protein expression analysis, the cells were collected at 24 h (n = 3).

### 2.7. Protein Extraction and Western Blot Analysis

HSCs as well as WT and VDR KO MSCs were cultured in uncoated 35-mm dishes. The cultured cells were washed with ice-cold PBS and treated with lysis buffer (Cell Signaling Technology, Catalog# 2881S, Danvers, MA, USA) for protein extraction. Following established protocols, cell lysates were collected and analyzed via western blotting, with subsequent quantification utilizing total protein measurements as previously described [32,50]. Briefly, protein samples were prepared and subjected to SDS-PAGE on Stain-free Gels with a 4–15% gradient (Bio-Rad, #4568084, Hercules, CA, USA). The separated proteins were transferred onto PVDF membranes (Bio-Rad #1704272). Subsequently, the membranes were incubated overnight at 4 °C with the α-SMA (1:3000), BMP6 (1:1000), and TGFβ3 (1:1000) primary antibodies diluted in TBST containing 10% non-fat milk. Following primary antibody incubation, membranes were treated with the peroxidase-conjugated anti-rabbit IgG secondary antibody diluted 1:5000 in TBST with 10% non-fat dry milk for 1 h. Detection and quantification of band intensities were performed using Image Lab 5.2.1 software in conjunction with ChemiDoc MP (Bio-Rad) [51]. Band intensities were normalized to total protein by calculating the ratio of each band’s intensity to the total protein intensity from the corresponding sample on the same blot.

### 2.8. Immunofluorescence Studies

Mouse eyes enucleated from euthanized mice were flash-frozen in liquid nitrogen, embedded in Tissue-Tek optimal cutting temperature (OCT) compound (Electron Microscopy Sciences, Hatfield, PA, USA), and cryosectioned for immunohistochemistry. 10 μm-thick Cryosections were fixed in 4% paraformaldehyde for 10 min and then blocked with 10% goat serum in 0.1% Triton X-100/PBS for 1 h at room temperature. Cryosections were incubated overnight at 4 °C with primary antibodies, including anti-α-SMA, anti-collagen III, anti-fibronectin, anti-FAM57B2, and anti-CD45, and diluted 1:200 in block solution. After washing with PBS three times for 10 min each, cryosections were incubated with goat anti-rabbit IgG (H + L) Alexa Fluor 488, donkey anti-Goat IgG (H + L) Alexa Fluor™ 555, goat anti-Mouse IgG (H + L) Alexa Fluor™ 633 secondary antibody at a dilution of 1:800 for 1 h and washed thoroughly with PBS. Examination of cryosections was performed using a Zeiss LSM 780 inverted laser-scanning confocal microscope (Zeiss Microscopy, Jena, Germany). Sections from 3 control and 3 treated corneas were examined. For cultured human and mouse fibroblasts, cells were cultured in 24-well cell culture plates with glass coverslips. The immunofluorescence protocol was the same as for the cryosections with three biological replicates included for each experimental group. Secondary antibody negative controls were examined in all immunostaining experiments.

The expression levels of α-SMA, fibronectin, and collagen III were quantified by scoring fluorescence intensity throughout the corneal stromal layer in mouse corneal frozen section samples. For each experimental group—NaOH wounded WT, NaOH wounded VDR KO and unwounded WT mice—six frozen sections were analyzed, representing three corneas from three mice. Fluorescence scoring was performed independently by a qualified observer in a blinded manner. A modified Allred scoring system was applied, in which relative expression was classified as 0 (Low), 1 (Mid-Low), 2 (High-Mid), or 3 (High) based on the distribution and staining intensity of the biomarker of interest [52,53]. Statistical analysis of biomarker scores was performed using an unpaired t-test in GraphPad Prism (version 10.5.0, GraphPad Software, San Diego, CA, USA)

### 2.9. Mouse Cornea Organ Culture

To examine the effect of Vit D on mouse cornea TGF β1-stimulated α-SMA expression, mouse corneas were placed in a culture dish in DMEM serum-free medium for a total 48 h and treated with TGF β1 (10 ng/mL) only, TGF β1 plus 1,25 Vit D (20 nM) or 24,25 Vit D (100 nM) (n = 5). Mouse corneas were incubated at 37 °C with 5% CO_2_. Total protein was prepared from corneal tissue using protein lysis buffer.

### 2.10. Real Time PCR

Total RNA was extracted from HSCs treated with TGF β1 plus 1,25 Vit D (20 nM) or 24,25 Vit D (100 nM) using the TRIzol protocol (ThermoFisher Scientific, # 15596026). Real time PCR was used to quantify TGFβ3, BMP2, BMP6, and BMPR2 mRNA levels. RPS19BP1was used as the internal RNA control. RT-PCR primers were generated from the PrimerBank database using National Center for Biotechnology Information (NCBI) sequence identification numbers (NM_003239.4, NM_001200.4, NM_001718, NM_001204.5, NM_194326.2 for TGF β3, BMP2, BMP6, BMPR2, and RPS19BP1, respectively). Primer sequences are listed in Table 1. mRNA was isolated and cDNA was synthesized using the Bio-Rad RT-PCR system. First-strand synthesis was performed at 42 °C for 60 min and inactivated at 85 °C for 5 min. cDNA was used for PCR amplification in triplicate using SYBR probes on the Bio-Rad system. Amplification was carried out at 95 °C for 10 min, followed by 40 cycles at 95 °C for 10 s and 60 °C for 30 s. Quantitative values were determined from the quantification cycle (Cq), and each sample was normalized using ∆Cq. The results were analyzed with the 2(∆∆Cq) method. Bio-Rad CFX Manager 3.1 software was used for RT-PCR data analysis.

### 2.11. Effects of VDR KO on Corneal Transparency and Recruitment of Immune Cells in NaOH Wounded Mice

To determine how VDR KO effects mouse corneal transparency following an alkali wound, wild-type (WT) C57BL/6J VDR KO mice (n = 5 per group) between 8 and 9 weeks old were anaesthetized with ketamine–xylazine. A central cornea injury was created by placing a 0.33 N NaOH soaked filter paper disc (2 mm diameter) on the central cornea for 10 s. Corneas were rinsed with 10 mL PBS and treated with topical antibiotic ointment 2 times per day for 3 days. Mice were treated with buprenorphine SR as an analgesic. Mouse corneal transparency was observed and photographed up to 21 days after wounding using a Topcon slit lamp (SL-D4).

To determine the effects of VDR KO on immune cell recruitment, wounded and control mouse corneas were collected 3 weeks after NaOH wounding (n = 5 per group) and immunostained with an anti-CD45 antibody. Briefly, mouse eyes were fixed in Zamboni fixative for 75 min and washed three times with PBS (5 min each). Corneas were excised along the sclera-corneal border, rehydrated in Triton X-100/PBS, and blocked with 10% goat serum and 0.1% Triton X-100 in PBS for 60 min at room temperature. They were then incubated with anti-CD45 antibody (1:200, Abcam) in 5% goat serum and 0.1% Triton X-100/PBS for 24 h at 4 °C with constant shaking. After three 10-min PBS washes, corneas were treated with Alexa Fluor 594 goat anti-rabbit IgG (1:1000) for 24 h at 4 °C and thoroughly washed in PBS. To image the corneas, each was flattened by making four radial cuts and mounted on a slide with the epithelium side facing up. CD45+ cell numbers were quantified using the Imaris Spots package (Oxford Instruments, Abingdon, UK). This 3D/4D visualization software effectively counts individual cells in 2D images. By inputting a 2D image and calibrating the Spots function—selecting the average cell diameter and setting initial sensitivity—the software labels cells as spots and reports the total count. A conservative approach was used to manually adjust counts, ensuring only clear cells (spots) in saturated areas were included [40].

### 2.12. Statistical Analysis

Data comparisons between two groups were conducted using an unpaired Student’s *t*-test. One-way ANOVA with Dunnett’s multiple comparison test was used when there was more the 1 experimental group. Statistical significance was determined based on a *p*-value of less than 0.05. Data analysis was performed using Graph Pad Prism 10.

## 3. Results

### 3.1. Effect of Vit D on α-SMA Protein Expression in the Human Stromal Cell Line and Primary HSC Cells

**Figure 1:**
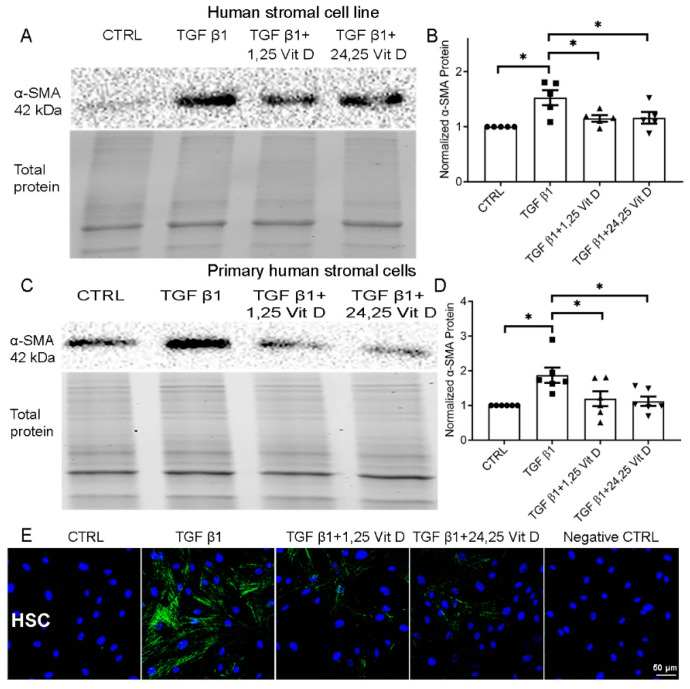
Western blot assay and immunostaining illustrating the effect of Vit D on α-SMA expression in the human stromal cell line and primary human stromal cells. Representative western blots from (**A**) the human stromal cell line and (**C**) HSCs treated with 1,25 Vit D and 24,25 Vit D. Corresponding average blot density graphs with total protein normalization from (**B**) the human stromal cell line and (**D**) HSCs treated with 1,25 Vit D and 24,25 Vit D. (**E**) Representative immunofluorescence labelling examining the effect of Vit D on α-SMA (green) expression in HSCs with representative secondary antibody negative control (n = 5, Mean ± SE. * *p* < 0.05; DAPI (blue)). Individual data points superimposed on bar graphs. Western blot original images are shown in Supplementary Materials Figure S1.

TGF β1-stimulated α-SMA protein expression was significantly decreased after 72 h in the human stromal cell line and primary HSCs treated with 1,25 Vit D or 24,25 Vit D (Figure 1A–D, Supplementary Figure S1). 1,25 Vit D reduced TGF β1–stimulated α-SMA by 52.3% ± 4.1% (*p* < 0.001) in human stromal cells, while 24,25 Vit D reduced TGF β1–stimulated α-SMA by 48.7% ± 3.9% (*p* < 0.001) (Figure 1B,D). Figure 1E demonstrates decreased α-SMA immunostaining following 1,25 Vit D or 24,25 Vit D treatment in HSCs, highlighting the potential of Vit D metabolites to inhibit myofibroblast differentiation.

### 3.2. Effect of Vit D on α-SMA Protein Expression in Primary WT and VDR KO MSCs

**Figure 2:**
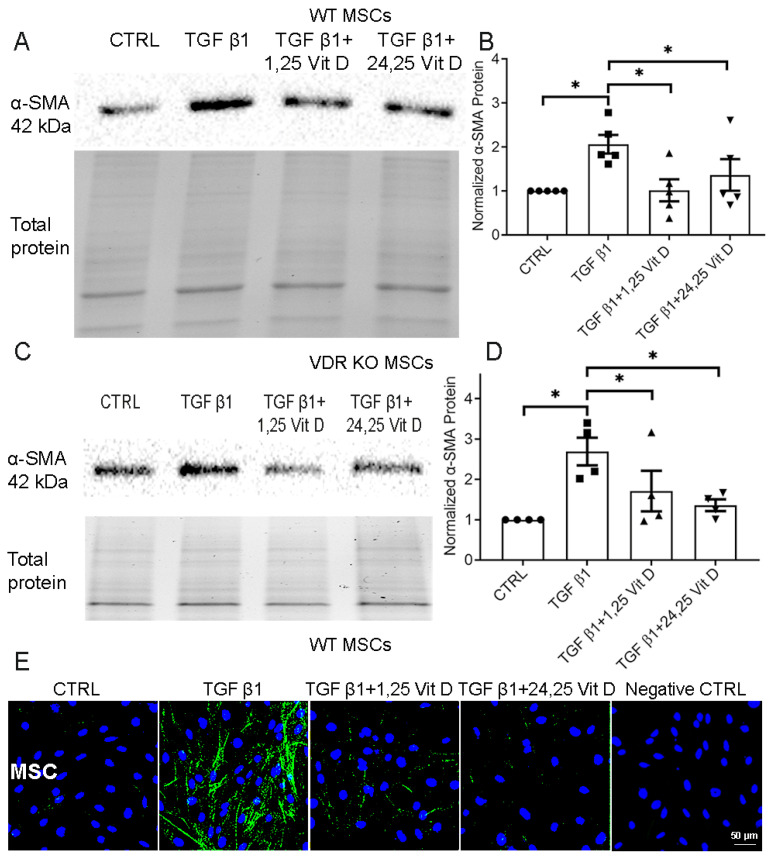
Western blot assay examining the effect of Vit D on α-SMA expression in MSCs and VDR KO MSCs. Representative western blots from (**A**) MSCs and (**C**) VDR KO MSCs treated with 1,25 Vit D and 24,25 Vit D. Corresponding average blot density graphs with total protein normalization from (**B**) MSCs and (**D**) VDR KO MSCs treated with 1,25 Vit D and 24,25 Vit D. (**E**) Representative immunofluorescence labelling examining the effect of Vit D on α-SMA expression (green) in MSCs with representative secondary antibody negative control (n = 5, Mean ± SE. * *p* < 0.05; DAPI (blue)). Western blot original images are shown in the Supplementary Materials Figure S2.

TGF β1-stimulated α-SMA protein expression was decreased in cultured WT (Figure 2A,B) and VDR KO MSCs (Figure 2C,D, Supplementary Figure S2) 72 h after 1,25 Vit D or 24,25 Vit D exposure (*p* < 0.05). Unstimulated α-SMA protein expression was not significantly different between WT MSCs and VDR KO MSCs. Figure 2E demonstrates decreased α-SMA immunostaining following 1,25 Vit D or 24,25 Vit D treatment in MSCs.

### 3.3. Effect of Vit D on TGF β1-Stimulated α-SMA Protein Expression in Mouse Corneas

**Figure 3:**
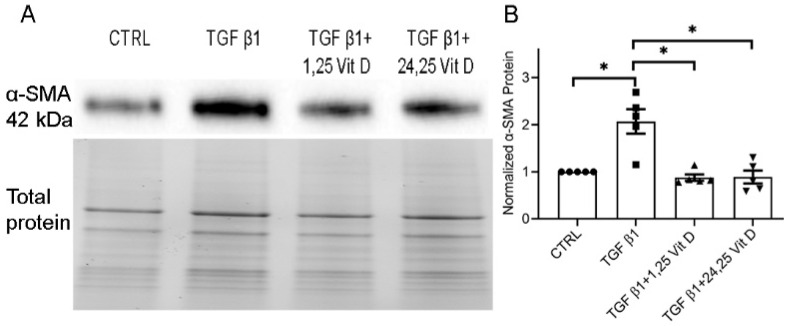
Western blot results examining the effect of Vit D on α-SMA protein expression in mouse corneal tissue. (**A**) Representative western blots from mouse corneal tissue treated with 1,25 Vit D and 24,25 Vit D. (**B**) Corresponding average blot density graph, normalized to total protein, of mouse corneal tissue treated with 1,25 Vit D and 24,25 Vit D (n = 5, Mean ± SE. * *p* < 0.05). Individual data points superimposed on bar graphs. Western blot original images are shown in the Supplementary Materials Figure S3.

Cultured mouse corneas were treated with TGF β1, with or without 1,25 Vit D or 24,25 Vit D, in DMEM serum-free medium. Figure 3 (and Supplementary Figure S3) shows significantly increased α-SMA protein expression in TGF β1-treated corneas compared to controls, and decreased α-SMA protein expression in mouse corneas treated with TGF β1 plus 1,25 Vit D or 24,25 Vit D.

### 3.4. Effect of Vit D on Antifibrotic Protein Factor mRNA Expressional Levels in HSCs

**Figure 4:**
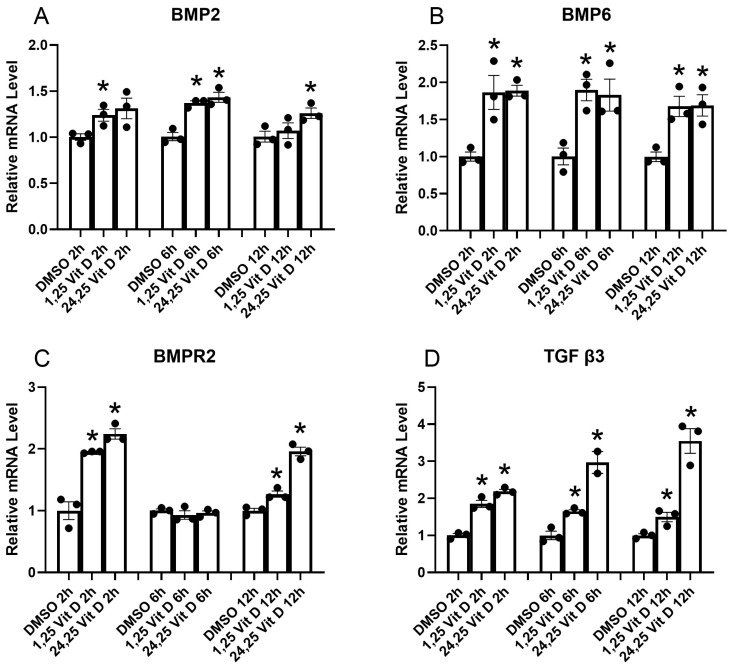
HSCs qPCR results for BMP2, BMP6, BMP2R, and TGFβ3 at indicted time points following 1,25D and 24,25D exposure. * *p* < 0.05 compared to DMSO vehicle, n = 3.

TGF-β family members, including BMPs, are key regulators of tissue repair and fibrosis. BMP2 and BMP6 are notable antifibrotic factors, with BMP2 demonstrating the ability to inhibit fibrogenic signaling pathways and reduce collagen deposition in various tissues. Figure 4 shows qPCR results demonstrating the influence of Vit D on mRNA levels of several antifibrotic protein factors in HSCs. Results demonstrate that BMP2, 6, and BMPR2 mRNA, along with that of TGF β3, are significantly elevated by both 1,25 Vit D and 24,25 Vit D at most of the time points tested.

### 3.5. Effect of Vit D on BMP6 and TGF β3 Protein Expression in HSCs

**Figure 5:**
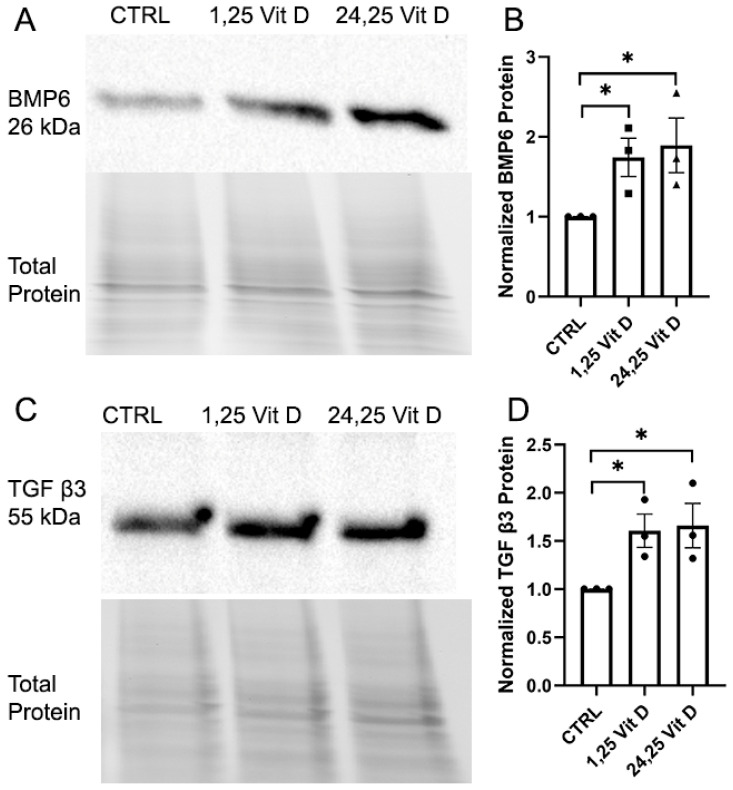
Representative western blots with accompanying density plots (relative %) demonstrating the effects of 1,25 Vit D and 24,25 Vit D on HSC (**A**,**B**) BMP6 and (**C**,**D**) TGF β3 protein expression; * *p* < 0.05 by unpaired *t*-test. Dots represent individual data points. Western blot original images are shown in the Supplementary Materials Figure S5.

Figure 5 (and Supplementary Figure S4) shows western blot data demonstrating increased HSC Vit D-stimulated protein levels of BMP6 (A,B) and TGF β3 (C,D), which were selected for protein measurement following the qPCR experiments. Treatment with 24,25 Vit D significantly increased both BMP6 and TGF-β3 protein levels compared with the control group. BMP6 expression showed an approximately 1.9 ± 0.3 fold elevation, while TGF-β3 expression increased by about 1.7 ± 0.2 fold. 1,25 Vit D also enhanced BMP6 and TGF-β3 expression, but to a slightly lesser extent. 1,25 Vit D-treated HSCs showed an approximately 1.7 ± 0.2 fold BMP6 increase, and TGF-β3 expression rose by roughly 1.6 ± 0.2 fold. These results suggest that both active vitamin D metabolites upregulate BMP6 and TGF-β3.

### 3.6. Effect of VDR KO on Corneal Fibrosis

**Figure 6:**
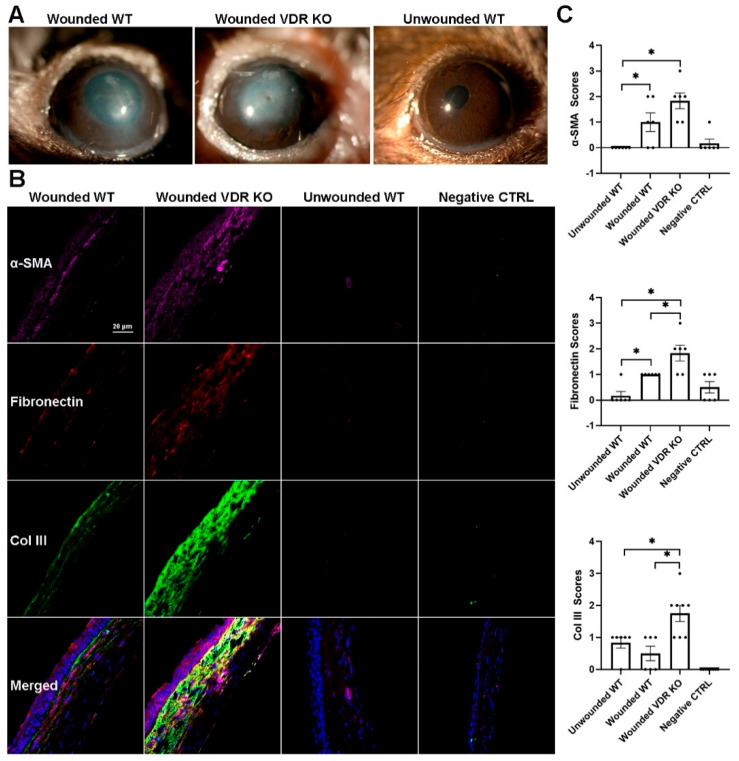
(**A**) Representative slit lamp images showing the effects of alkali wounding (0.33N NaOH) on WT, VDR KO corneas 10 days after wounding along with an unwounded WT cornea. (**B**) Representative α-SMA (magenta), fibronectin (orange), and collagen III (green) protein expression in WT and VDR KO mice 14 days after NaOH wounding along with an unwounded WT cornea and secondary antibody negative controls (DAPI-blue). (**C**) Blinded scoring of protein expression levels of the proteins and groups represented in (**B**). Data are presented as mean ± SEM; * *p* < 0.05 by unpaired *t*-test. Dots represent individual data points.

**Figure 7:**
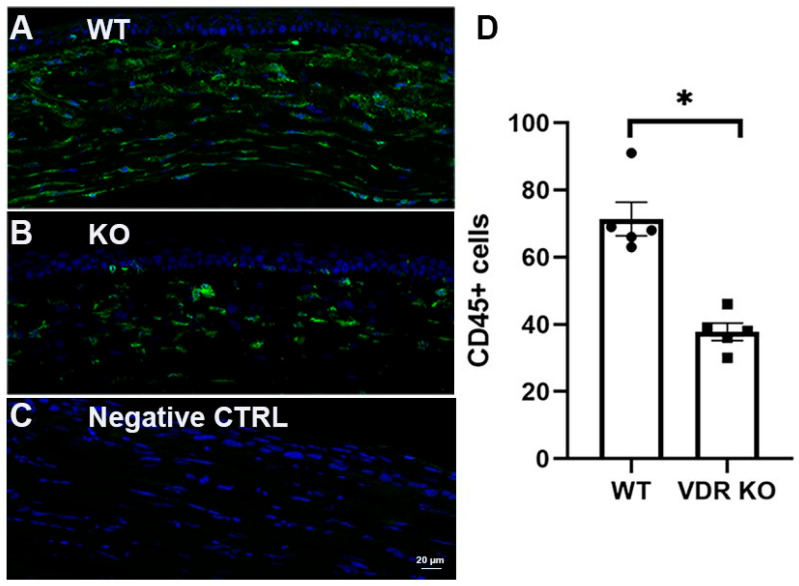
CD45 immunostaining (green) of NaOH wounded (0.33N) (**A**) WT and (**B**) VDR KO mouse corneas 3 weeks after wounding. (**C**) Secondary antibody negative control. DAPI staining in blue. (**D**) CD45+ cell count in WT and VDR KO corneas with individual data points superimposed. * *p* < 0.05.

WT and VDR KO mouse corneas were injured using a 0.33N NaOH-soaked filter disc applied to the cornea for 10 s. Corneal healing/fibrosis was monitored with a slit lamp, with fluorescein staining utilized to examine epithelial healing. Epithelium of all eyes was healed by day 5; however, VDR KO mouse corneas exhibited more haze compared to WT corneas throughout the healing process. Figure 6A shows representative images of WT and VDR KO mouse corneas 14 days post-injury, with the VDR KO cornea displaying both a central epithelial defect and greater opacity. Figure 6B shows representative immunostaining for α-SMA, fibronectin, and collagen III in WT and VDR KO corneas, respectively, 14 days after alkali wounding, revealing increased expression of all three fibrotic marker proteins in the VDR KO versus WT corneas. Figure 6C shows the scoring results for each protein’s immunofluorescence. Following wounding, WT corneas showed significantly increased α-SMA and fibronectin expression (*p* < 0.05) with no increase in collagen III expression. All three proteins were significantly increased in VDR KO corneas compared to unwounded WTs, with fibronectin and collagen III levels significantly increased in VDR KO compared to wounded WT corneas. The mean α-SMA score was elevated compared to wounded WTs but did not reach significance. In addition, the number of CD45+ immune cells was significantly reduced in VDR KO corneas compared to WT corneas three weeks post-wounding (Figure 7). Specifically, wounded VDR KO corneas had 68% fewer CD45+ cells than WT post-injury (*p* < 0.001). This impaired immune recruitment likely potentiates fibrosis.

### 3.7. Localization of FAM57B in the Mouse Cornea

**Figure 8:**
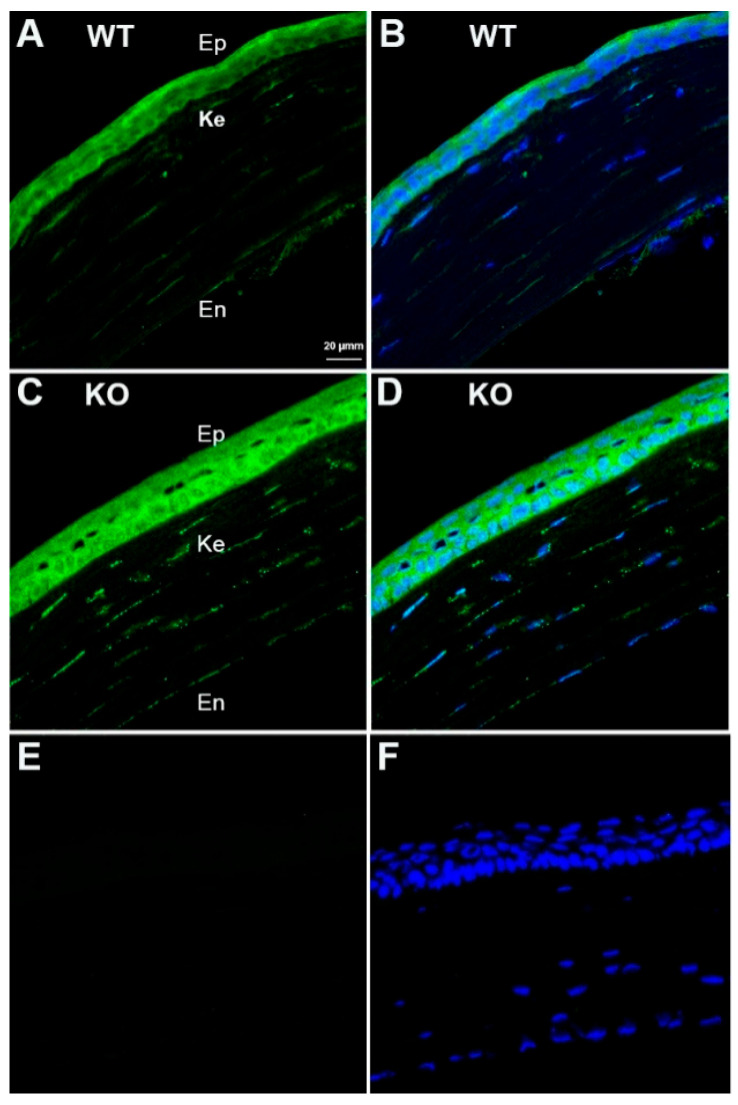
FAM57B immunostaining (green) in mouse corneas. (**A**,**B**) WT corneas with and without DAPI staining (blue). (**C**,**D**) KO corneas with and without DAPI staining. (**E**,**F**) Secondary antibody negative control WT corneas with and without DAPI staining. Abbreviations: Ep-epithelium, Ke-keratocytes, En-endothelium.

The FAM57B protein was recently described as a 24,25D receptor [31]. Immunohistochemical labeling of WT and KO mouse corneas revealed the presence of FAM57B in all 3 corneal cell types in both genotypes, with no discernable differences in labelling intensity (Figure 8).

## 4. Discussion

Vit D is well-known for its role in bone health and calcium metabolism. 1,25 Vit D is a central regulator of calcium and phosphate homeostasis and promotes cellular differentiation, stimulates apoptosis, and influences the proliferation of selected cell types. Vit D is also increasingly recognized for protective effects in head and neck tissues and cancers [54]. It modulates both innate and adaptive immunity by promoting antimicrobial peptides like cathelicidin and defensins [55]. Vitamin D also helps regulate autoimmune responses by suppressing pro-inflammatory T cells and enhancing regulatory T cells, with relevance to diseases like multiple sclerosis and rheumatoid arthritis [56]. Approximately 30 min of daily sun exposure without sunscreen is essential for the body’s natural production of vitamin D. During this process, UVB rays convert 7-dehydrocholesterol in the skin to vitamin D3, which is then hydroxylated in the liver and kidneys to form calcitriol [57].

Vitamin D plays a significant role in supporting ocular health. In the eye, vitamin D helps reduce cytokine production, oxidative stress, and microglial activation, thereby protecting against diseases such as age-related macular degeneration, dry eye syndrome, and diabetic retinopathy [14,58]. We previously determined that exposing cultured corneal epithelial cells to UV-B, in the presence of 7-dehydrocholesterol, resulted in a UV-B-dose dependent increase in vitamin D3, 1,25 Vit D and 24,25 Vit D. That same study determined that dietary vitamin D supplementation in rabbits resulted in elevated tear and anterior chamber 1,25 Vit D and 24,25 Vit D levels [58].

Vit D’s protective action is a cornerstone of numerous diseases, with Vit D deficiency-initiated oxidative stress being a significant contributor [59]. In cardiovascular tissues, VDR is expressed in fibroblasts, vascular endothelial cells, and cardiomyocytes, where vitamin D reduces renin–angiotensin system activity, enhances ACE2 expression, and lowers pro-inflammatory cytokines such as IL-1β and TNF-α [59]. Several studies have investigated the relationship between Vit D and fibrosis in different organs such as the liver, lungs, and heart [4,5,60,61]. Vit D can exert potent antifibrotic effects by attenuating NLRP3 assembly and reducing IL-1β release in both hepatic and cardiovascular tissue [59,62,63]. 1,25 Vit D was shown to prevent TGF β1-induced human cardiac fibroblast activation by inhibiting SMAD2 phosphorylation [60]. In the respiratory system, Vit D inhibits bleomycin and TGF β-induced pulmonary fibrosis [64,65]. The current study indicates that TGF β1-induced cornea myofibroblast differentiation, as determined by examining α-SMA expression, was reduced by 1,25 Vit D and 24,25 Vit D. Importantly, it was also found that VDR KO led to more severe NaOH-induced corneal fibrosis as compared to WT mice. A more comprehensive assessment, including additional myofibroblast markers (such as fibronectin, collagen type III, and others), as well as broader transcriptional profiling, would provide a more robust evaluation of the myofibroblast differentiation process.

Wounded corneas from VDR KO mice exhibited significantly increased haze compared to those from WT mice, indicating enhanced fibrotic remodeling. Immunohistochemical analysis revealed elevated expression of key fibrotic markers in the wounded VDR KO corneas, including α-SMA, fibronectin, and collagen III. The trend of increased α-SMA expression suggests a greater presence of myofibroblasts. Similarly, increased fibronectin and collagen III deposition reflect heightened extracellular matrix production, further contributing to stromal disorganization and haze formation. These findings suggest that VDR signaling plays a critical regulatory role in limiting fibrotic responses during corneal wound healing.

Although the exact mechanisms are not fully understood, previous studies suggest that Vit D’s antifibrotic effects can be mediated by regulation of specific signaling pathways involved in fibrosis, modulation of the immune system, and its anti-inflammatory properties. Vit D metabolites may suppress TGF-β/Smad signaling. TGF β3 has been shown to be an antifibrotic agent in the cornea and several other tissues [66,67,68,69,70], possibly by upregulating SMAD7 in the cornea [71]. The current study demonstrates increased TGF β3 mRNA and protein expression in HSCs following exposure to both 1,25 Vit D and 24,25 Vit D; thus, it is possible that Vit D may reduce corneal fibrosis through the TGF β3 pathway.

Another group of TGF protein superfamily members with anti-fibrotic properties are the BMPs. A host of BMPs have been studied in the cornea, including BMP-2, -6, and -7, with each subtype being described as antifibrotic [12,13,72,73,74,75,76]. BMP-6 and BMPR2 have been reported to mediate phenotypic reversal of corneal myofibroblasts to keratocytes [77], and BMP-7 has been shown to attenuate corneal fibrosis [78,79,80]. Vit D and BMP-2 act synergistically to promote osteogenic differentiation, and PDIA3, a proposed alternative Vit D receptor, seems to be involved in this response [81,82]. The current study demonstrates increased HSC mRNA expression of BMP-2, -6, and BMPR2 (and BMP-6 protein) following 1,25 Vit D and 24,25 Vit D exposure. The influence of 24,25 Vit D on BMP signaling had not previously been reported in any cell type. The current study did not examine the possibility that the BMPs or TGF β3 were affected by TGF β1. A separate study found that Vit D supplementation of Vit D-insufficient diabetic periodontitis patients resulted in elevated BMP-2 serum and gingival crevicular fluid samples [83]. Another study demonstrated increases of all 3 TGFβ isoforms following Vit D treatment of dermal fibroblasts [84]. In addition to BMP superfamily members, additional transcription factors and proteins that could be involved in Vit D antifibrotic activity include, but are not limited to, Snail, Slug, ZEB1, Twist, S100A4, and MYH11. To the best of our knowledge, the influence of Vit D on expression of these transcription factors and proteins has not previously been examined, and additional studies will be needed to determine how they are influenced by Vit D in the cornea. Vitamin D upregulation of BMPs could potentially counteract TGF-β–induced fibrosis, as BMP-7 inhibits TGF β1–driven myofibroblast differentiation in renal and corneal models [85]. It would be beneficial for future studies to determine if Vit D metabolites directly modulate SMAD7 (an inhibitor of TGF-β signaling) or BMPR2 transcription.

It is well known that 1,25 Vit D works primarily through a VDR-mediated transcription mechanism in which the hormone directly regulates gene expression of a wide variety of Vit D-dependent genes in Vit D target cells. A recent study demonstrated that VDR ligands inhibit hepatic stellate cell activation and fibrosis, that VDR KO mice spontaneously develop liver fibrosis, and that VDR antagonizes SMAD3/TGF β1 activation of profibrotic genes [4]. Results of the current study determined that TGF β1-stimulated α-SMA protein expression was significantly reduced in both cultured WT and VDR KO MSCs following exposure to 1,25 Vit D or 24,25 Vit D. Our group previously described significant physiological effects of 1,25 Vit D and 24,25 Vit D in VDR KO corneal epithelial cells, and attributed those findings to the presence of alternative Vit D receptors in these cells [32,33], and in particular the protein disulfide isomerase family member 3 (Pdia3), which has been identified as an alternative membrane-associated Vit D receptor [86]. To that end, we previously demonstrated Pdia3 expression in mouse and human corneal keratocytes [46]. Although its role beyond binding 24,25 Vit D is unexplored, we determined that a 24,25 Vit D receptor protein recently identified by St-Arnaud’s group, FAM57B2 [31], is also present in mouse corneal keratocytes as well as mouse corneal epithelial and endothelial cells. We attribute the current results observed in KO mouse MSCs to the presence of Pdia3, FAM57B2, or another alternative Vit D receptor in VDR KO MSCs. Future work could determine if FAM57B knockdown/knockout would abolish 24,25 Vit D’s antifibrotic effects.

It is important to compare results obtained from mouse and human studies, particularly when describing mouse models of human disease and developing or describing potential therapeutics. The current study found that TGF β1-induced corneal myofibroblast differentiation was significantly reduced by 1,25 Vit D and 24,25 Vit D in the human corneal stromal cell line, HSCs, MSCs, and cultured mouse corneas. These effects were confirmed in wounded mouse corneas. A previous study from our group in the corneal epithelium revealed species differences in Vit D metabolism and cell proliferation between humans and mice [46]. In cultured mouse corneal epithelium, 1,25 Vit D treatment increased CYP24A1, the enzyme responsible for inactivating 1,25 Vit D and forming 24,25 Vit D, decreased CYP27B1, the enzyme that activates Vit D, and did not affect proliferation. In human corneal epithelium, 1,25 Vit D also increased CYP24A1 expression but did not affect CYP27B1, and it reduced cell proliferation. In stromal cells of both species, 1,25 Vit D increased expression of both CYP24A1 and CYP27B1, highlighting species and cell type differences. The differences suggest divergent metabolic regulation, impacting potential therapeutic dosing. Human corneal fibroblasts may require higher Vit D concentrations for efficacy due to catabolic differences. Topical formulations (e.g., nanocarriers) could overcome bioavailability issues [87]. A limitation of this study is that while multiple donors were used to obtain the human primary fibroblasts, the cells from individual donors were not tracked in the cell culture studies and all male donors were used. The relatively small variability in the results would indicate that there was not tremendous variability between donors.

It was surprising that the in vitro data demonstrated that both 1,25 Vit D and 24,25 Vit D suppressed α-SMA expression in TGF β1-induced stromal cells, even in the absence of VDR (indicating VDR-independent antifibrotic effects), while the in vivo results demonstrated exacerbated fibrosis in VDR KO mice after alkali injury compared to WT mice. There are several possible explanations for these seemingly contradictory results. While it might be expected that alternative receptors and endogenous 24,25 Vit D might compensate for loss of VDR in the KO mice, it is not known what concentration of 24,25D is reaching the cornea in these mice or what the density of alternative Vit D receptors might be within in vivo keratocytes. In addition, the traditional VDR has a much higher affinity for 1,25 Vit D than other Vit D metabolites, thus it is likely that it would be difficult to overcome loss of VDR in an in vivo environment. While beyond the scope of this study, it would be interesting to determine if adding exogenous Vit D to in vivo KO mouse corneas would result in suppression of the observed fibrosis. These results also highlight the likely role of systemic and immune-mediated factors. Specifically, the reduced infiltration of CD45+ immune cells in wounded VDR KO corneas suggests impaired immune modulation—a key regulator of fibrosis resolution that cannot be replicated in isolated cell cultures. Thus, the in vivo phenotype likely stems from compounded effects: compromised systemic immune responses due to VDR deficiency, in conjunction with cell-autonomous stromal signaling via alternative receptors like FAM57B. Future studies dissecting cell-type-specific contributions (e.g., stromal vs. immune cells) should help to further clarify these pathways.

Modulating immune responses and reducing inflammation are critical for corneal repair; for example, ocular microbiota has been found to play a role in maintaining corneal health [88]. Moreover, Vit D was found to improve wound healing and reduce IL6 production in Staphylococcus aureus-infected human keratinocytes [89]. CD45 is a marker for all hematopoietic cells except for mature erythrocytes and platelets. In the normal human and mouse cornea, there are a significant number of resident CD45+ cells within the pericentral and central region [37,90,91]. After a corneal scrape wound, neutrophils extravasate from the limbal vessels and migrate through the corneal stroma to the site of damage [92,93]. By 48 h, neutrophil levels within the stroma are near those found before injury [94]. Previous studies demonstrated that CD45+ immune cell immigration within wounded corneas occurs in two waves during the first 48 h [94]. Corneal re-epithelialization is significantly delayed in neutropenic animals, and early leukocyte emigration appears to promote re-epithelialization [95]. The current study found reduced numbers of CD45+ immune cells in VDR KO vs. WT corneas three weeks after NaOH wounding. In addition, our group recently determined that CD45+ cell numbers are reduced in diabetic and VDR KO mouse corneas compared to normoglycemic mice, and that 24,25 Vit D increased the recruitment of CD45+ cells to diabetic mouse corneas after epithelial debridement [40]. It was previously found that the VDR is required for recruitment of macrophages in cutaneous wounds, with the number of macrophages in granulation tissue markedly reduced in VDR KO compared with WT mice [96]. In addition, hepatic neutrophils are also decreased in VDR KO mice. Despite an increased awareness of the presence of corneal inflammatory cells in recent years, data demonstrating their precise role in normal corneal homeostasis is still rare [97]. Our findings suggest that Vit D and the VDR may play an important role in mitigating corneal fibrosis by modulating the immune system.

CD45+ cells have been shown to be both beneficial and detrimental as related to fibrosis. For example, CD45 cell therapy was found to be beneficial for treating CCl_4_-induced liver fibrosis [98], and CD45+ cells were found to alleviate bleomycin-induced pulmonary fibrosis by limiting expansion and activation of group 2 innate lymphoid cells [99]. Alternative examples include CD45+ fibrocytes promoting corneal injury-induced fibrosis [100], and CD45(+) cells correlated with mitral valve fibrosis following myocardial infarction [101]. The present study demonstrated reduced CD45+ cell infiltration following NaOH injury in VDR KO mouse corneas, which showed an enhanced fibrotic response, indicating a possible beneficial role for these cells in preventing or resolving chemical injury-induced fibrosis.

Additional limitations of the current study include an absence of VDR antagonists for validation of cell culture studies and in situ and in vivo studies that did not incorporate the VDR KO mice, limited time points and Vit D concentrations examined, and lack of in vivo functional assessments for some of the studies. It is noteworthy that we consider the 1,25 Vit D and 24,25 Vit D concentrations tested in the current study (20 nM and 100 nM, respectively) to be pharmacological doses and not physiological concentrations. We previously determined that rabbit aqueous humor 1,25 Vit D and 24,25 Vit D levels to be as high as 0.04 nM and 0.27 nM, respectively (with no extra dietary Vit D supplementation) [58]. To the best of our knowledge, no studies have measured these Vit D metabolite concentrations in the eyes of humans or mice. We chose the higher 24,25 Vit D concentration (relative to the 1,25 Vit D concentration) due to its lower affinity for the VDR as compared to the high affinity agonist, 1,25 Vit D. The affinity of 24,25 Vit D to the alternative receptors has not been thoroughly studied. Those Vit D concentrations examined in the current study were the same as those used in all of our previous cornea Vit D studies.

The current study indicates that 1,25 Vit D and 24,25 Vit D can be beneficial for reducing fibrotic wound healing following corneal alkali injuries. Previous work from our group demonstrated that Vit D promotes corneal epithelial wound healing. VDR KO mice have significantly slower cornea epithelial wound healing rates as compared to controls, and Vit D deficient diabetic mice have slower corneal epithelial wound healing than wild-type diabetic mice. Topical 1,25 Vit D significantly accelerated corneal epithelial wound healing in normoglycemic, diabetic, and diabetic VDD mice, and topical 24,25 Vit D significantly accelerated corneal epithelial wound healing of diabetic VDD and VDR KO mice [40]. We also found significant roles for Vit D in maintaining corneal epithelial adherens junctions, promoting gap junction communication, and affecting cell migration and proliferation. Thus, 1,25 Vit D and 24,25 Vit D appear to promote corneal wound healing following both mechanical and chemical wounding.

## 5. Conclusions

Our group has previously demonstrated the positive influence of Vit D on corneal epithelial cells and its involvement in corneal wound healing. The current study focused on the influence of Vit D on corneal keratocyte wound responses. We demonstrated that VDR KO resulted in increased NaOH-induced corneal fibrosis. Conversely, exposure of cultured stromal cells and mouse corneas to Vit D metabolites (1,25 Vit D and 24,25 Vit D) significantly decreased expression of α-SMA, a hallmark of myofibroblast phenotype. Furthermore, both 1,25 Vit D and 24,25 Vit D significantly increased the expression of key TGF-β superfamily antifibrotic factors BMP2, BMP6, BMPR2, and TGF-β3 at both the mRNA and protein levels in human stromal cells. These findings highlight the robust antifibrotic potential of Vit D metabolites through modulation of critical signaling pathways. While both metabolites suppress fibrosis, 24,25 Vit D operates primarily through the newly identified receptor FAM57B (detected in all corneal layers), whereas 1,25 Vit D likely acts via VDR and PDIA3. 24,25 Vit D uniquely enhances BMP6/TGF-β3 signaling without VDR dependence, suggesting non-overlapping therapeutic targets.

Additionally, our results demonstrate that VDR plays a crucial role in immune cell recruitment during corneal wound healing. Specifically, VDR KO mouse corneas exhibited significantly reduced CD45+ immune cell infiltration following alkali injury compared to WT controls. Given the essential functions of CD45+ immune cells, including modulation of inflammation, clearance of debris and pathogens, secretion of cytokines and growth factors, and regulation of keratocyte activation, this impaired recruitment in VDR KO mice likely contributes to increased corneal fibrosis.

In summary, our findings support the beneficial role of Vit D and its receptor across multiple phases of corneal wound healing. Through the likely reduction of myofibroblast differentiation and enhancement of antifibrotic signaling through BMPs and TGF-β3 pathways, Vit D metabolites represent promising therapeutic agents for reducing corneal fibrosis after injury or surgery. The broader implications of this study along with our previous work demonstrating the beneficial effects of Vit D on corneal epithelial wound healing, including topical application of 1,25 and 24,25 Vit D, suggest potential clinical applications for topical Vit D analogs in treating ocular surface disorders characterized by fibrosis and impaired wound healing. Although this study lacks in vivo topical or systemic Vit D treatment—an area that should be prioritized in future fibrotic wound healing research—there is strong rationale to pursue such work. Given the beneficial aspects of Vit D for many aspects of corneal wound healing and physiology, future studies should test metabolite-specific Vit D formulations (e.g., 24,25 Vit D for VDR-independent action) for their anti-fibrosis and beneficial wound healing properties following corneal injuries.

## Figures and Tables

**Table 1:** Summary of primer pair sets.

Gene	Direction	Primer Sequence	Product Size (bp)	Melting Temperature
Homo sapiens TGFβ3	Forward	5′-AAGAAGCGGGCTTTGGAC-3′	62	60 °C
	Reverse	5′-CGCACACAGCAGTTCTCC-3′		
Homo sapiens BMP2	Forward	5′-CCCAGCGTGAAAAGAGAGAC-3′	168	61 °C
	Reverse	5′-GAGACCGCAGTCCGTCTAAG-3′		
Homo sapiens BMP6	Forward	5′-AGCGACACCACAAAGAGTTCA-3′	159	62 °C
	Reverse	5′-GCTGATGCTCCTGTAAGACTTGA-3′		
Homo sapiens RPS19BP1	Forward	5′-CGTGGCTGAGTCTGTGAGC-3′	120	60 °C
	Reverse	5′-GTCTTCCTCGGTGAACACG-3′		
Homo sapiens BMPR	Forward	5′-CTTTACTGAGAATTTTCCACCTCCTG-3′	90	59 °C
	Reverse	5′-GCCAAAGCAATGATTAT TGTCTCATC-3′		

## Data Availability

Any data not contained within the manuscript will be available on request.

## Supplementary Materials

The following supporting information can be downloaded at: https://www.mdpi.com/article/10.3390/cells14201583/s1, Figure S1. Western blot assay illustrating the effect of Vit D on α-SMA expression in the human stromal cell line and primary human stromal cells. Representative western blots and total protein stain-free images from (A) the human stromal cell line and (B) HSC treated with 1,25 Vit D and 24,25 Vit D. Figure S2. Western blot assay examining the effect of Vit D on a-SMA expression in MSC and VDR KO MSC. Representative western blots and total protein stain-free images from (A) MSC and (B) VDR KO MSC treated with 1,25 Vit D and 24,25 Vit D. Figure S3. Western blot results and total protein stain-free image examining the effect of Vit D on a-SMA protein expression in mouse corneas treated with 1,25 Vit D and 24,25 Vit D. Figure S4. Representative western blots and total protein stain-free images demonstrating the effects of 1,25 Vit D and 24,25 Vit D on HSC (A) BMP6 and (B) TGF β3 protein expression (n = 3).
