# Supplementary material for: Suppression of Cornea Stromal Fibrosis by Vitamin D
Source: Cells. 2025 Oct 11;14(20):1583. doi: 10.3390/cells14201583 (PMC12562390; doi:10.3390/cells14201583)
Supplement: Supplementary file 1 [file cells-14-01583-s001.zip › Supplemental Figs -Cells.pdf]

Figure S1. Western blot assay illustrating the effect of Vit D on α-SMA expression in the human stromal cell line and primary human stromal cells. Representative western blots and total protein stain-free images from (A) the human stromal cell line and (B) HSC treated with 1,25 Vit D and 24,25 Vit D.

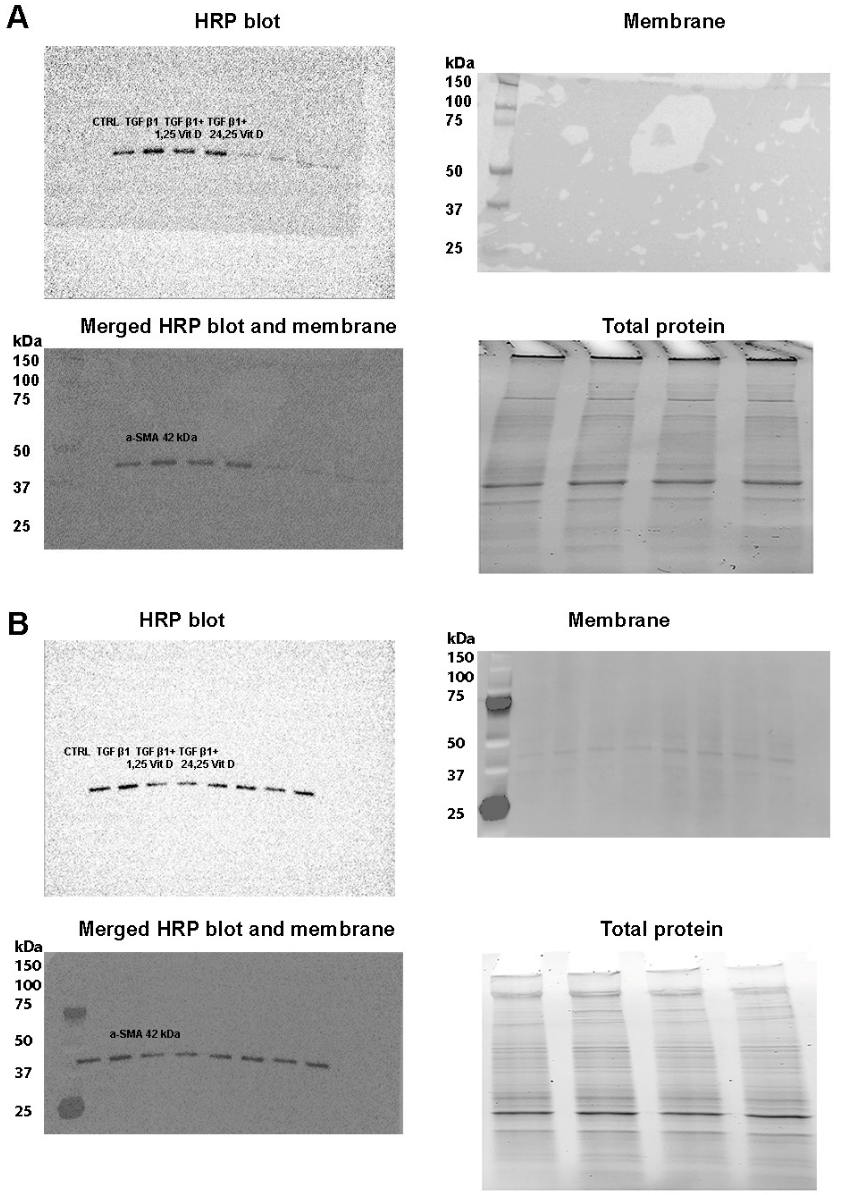

Figure S2. Western blot assay examining the effect of Vit D on α-SMA expression in MSC and VDR KO MSC. Representative western blots and total protein stain-free images from (A) MSC and (B) VDR KO MSC treated with 1,25 Vit D and 24,25 Vit D.

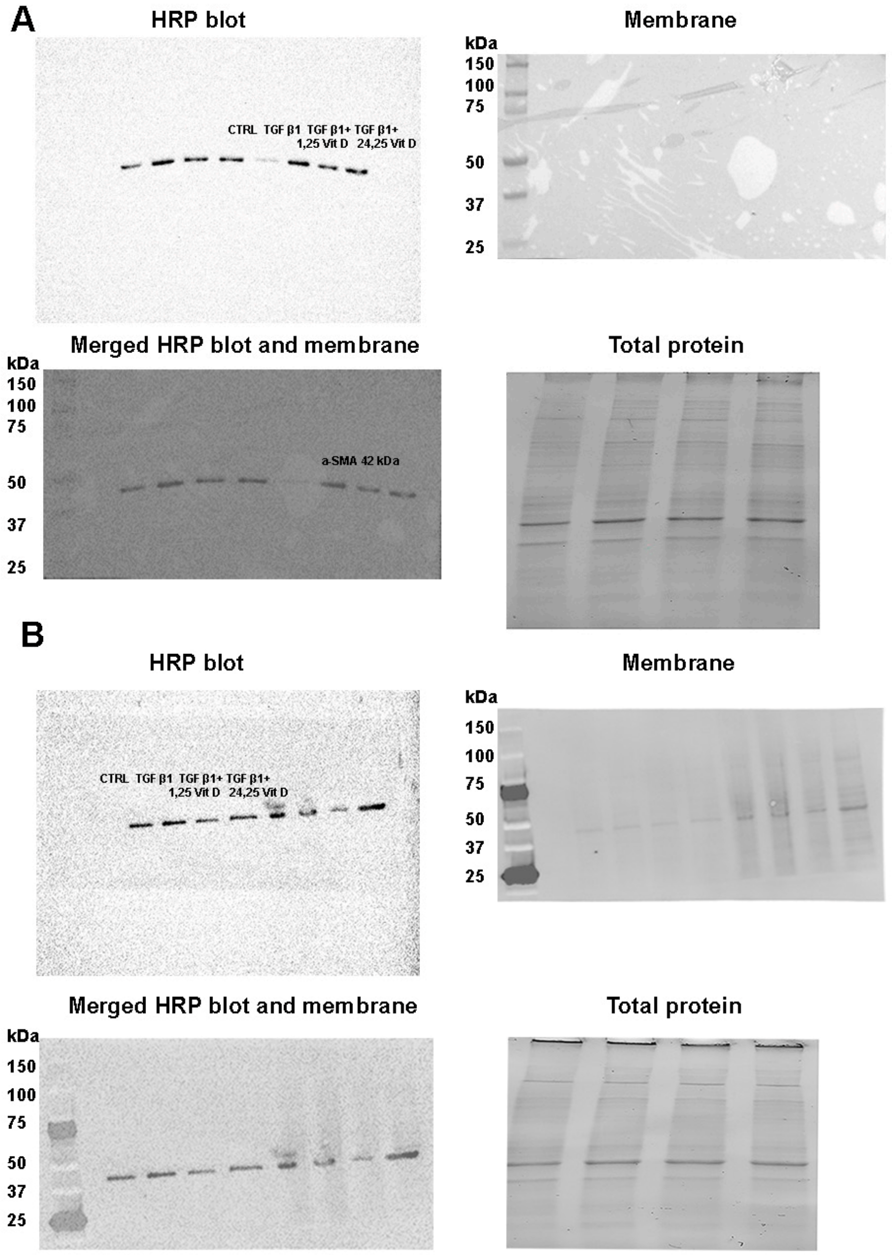

Figure S3. Western blot results and total protein stain-free image examining the effect of Vit D on α-SMA protein expression in mouse corneas treated with 1,25 Vit D and 24,25 Vit D.

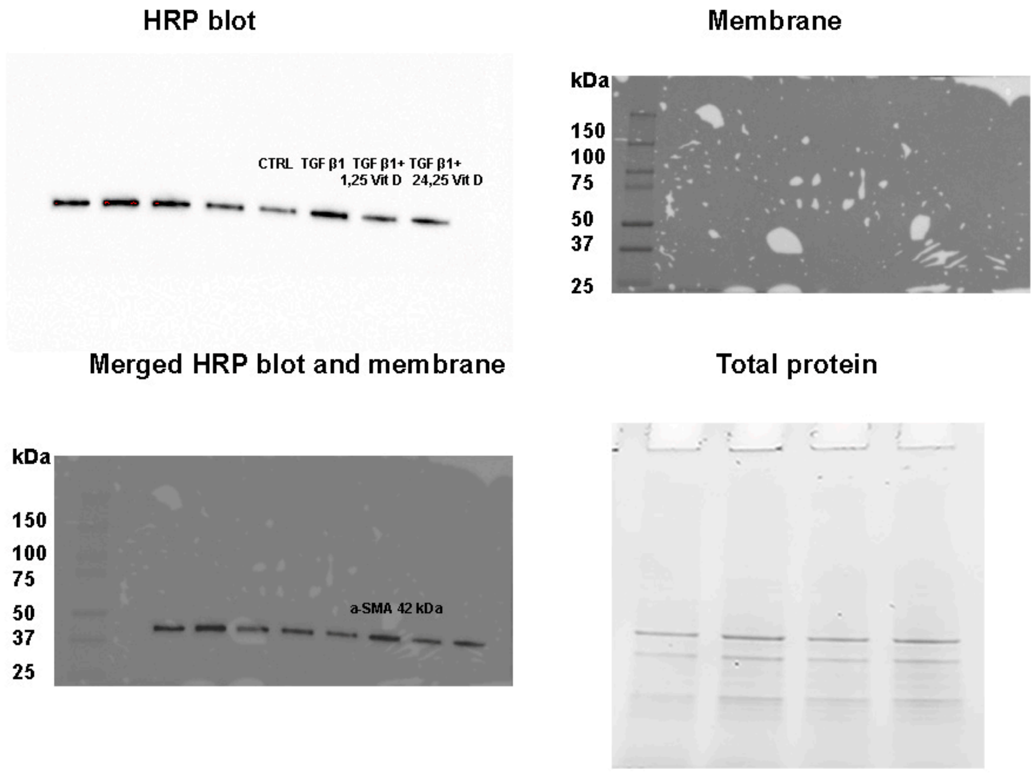

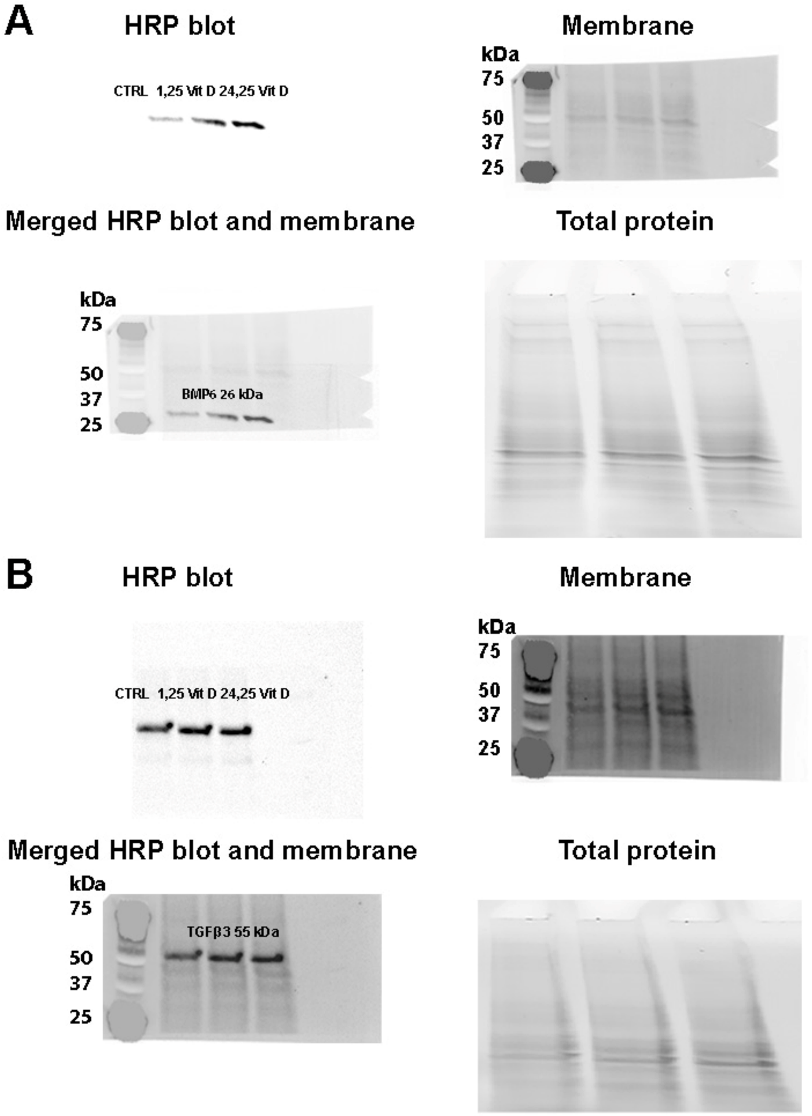

Figure S4. Representative western blots and total protein stain-free images demonstrating the effects of 1,25 Vit D and 24,25 Vit D on HSC (A) BMP6 and (B) TGF β3 protein expression (n=3).
