# Supplementary figures and images for: Suppression of Cornea Stromal Fibrosis by Vitamin D
Source: Cells. 2025 Oct 11;14(20):1583. doi: 10.3390/cells14201583 (PMC12562390; doi:10.3390/cells14201583)

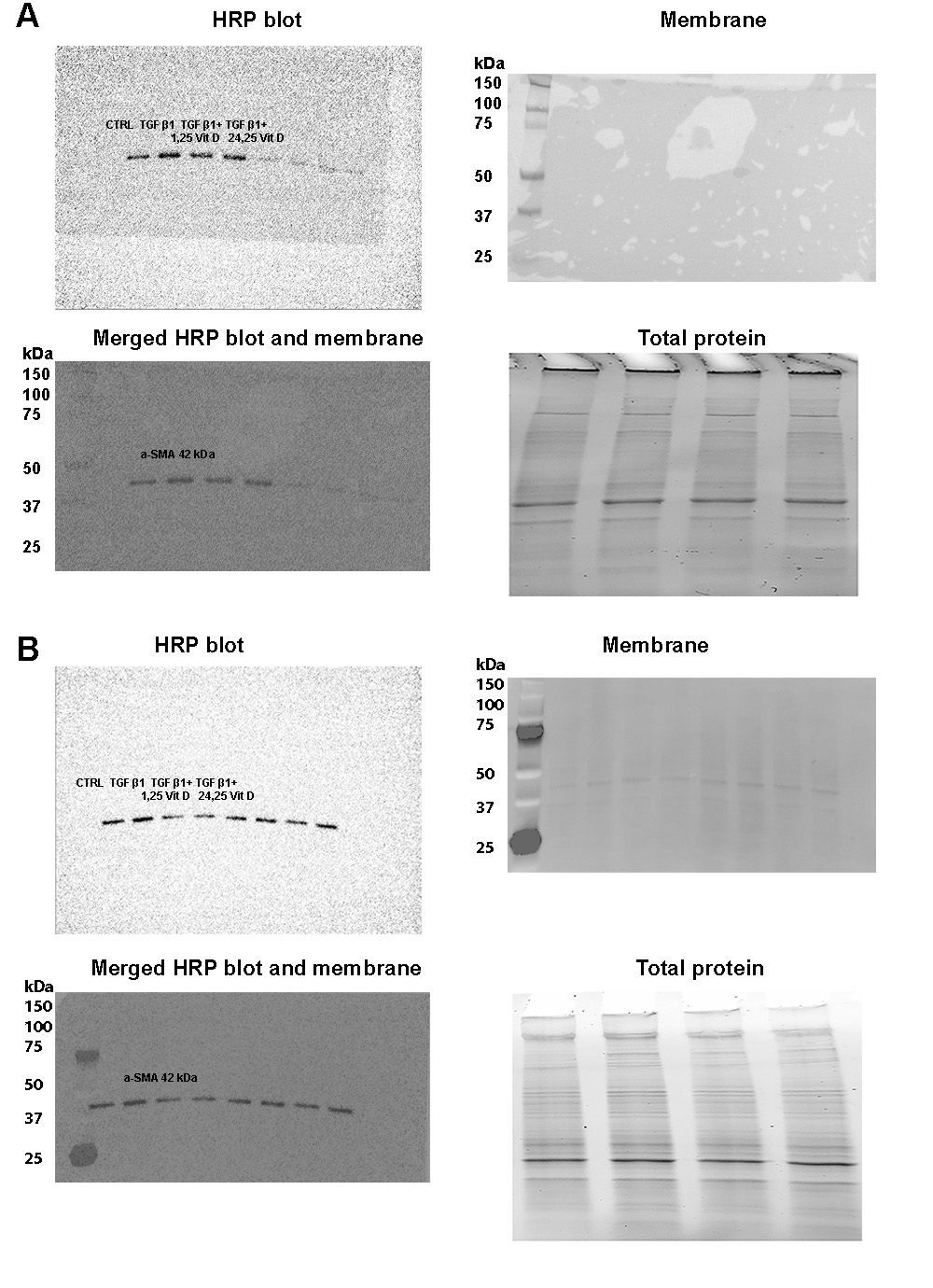

Supplement: Supplementary file 1 [file cells-14-01583-s001.zip › Figure S1.jpg]

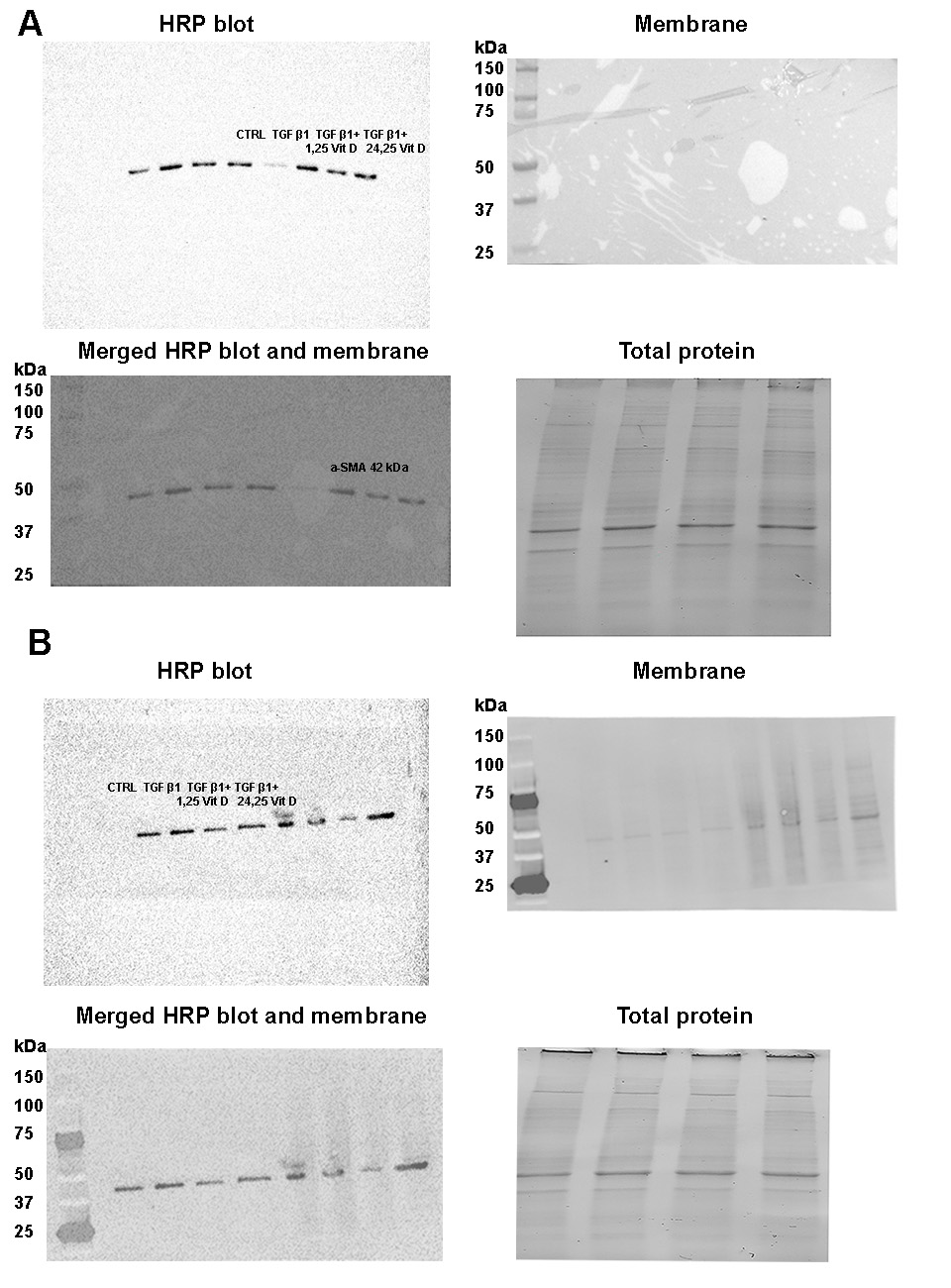

Supplement: Supplementary file 1 [file cells-14-01583-s001.zip › Figure S2.jpg]

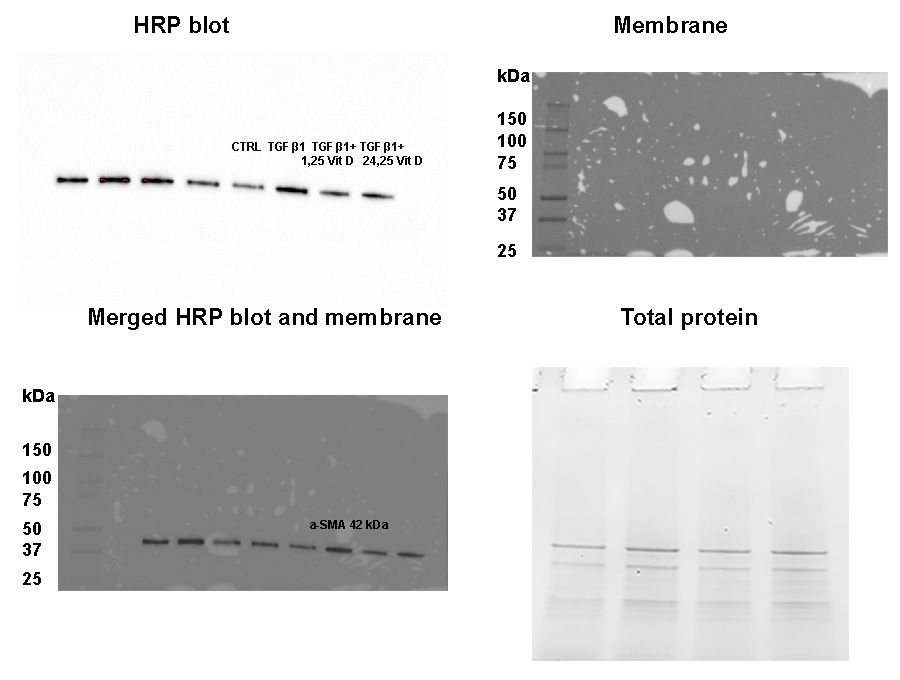

Supplement: Supplementary file 1 [file cells-14-01583-s001.zip › Figure S3.jpg]

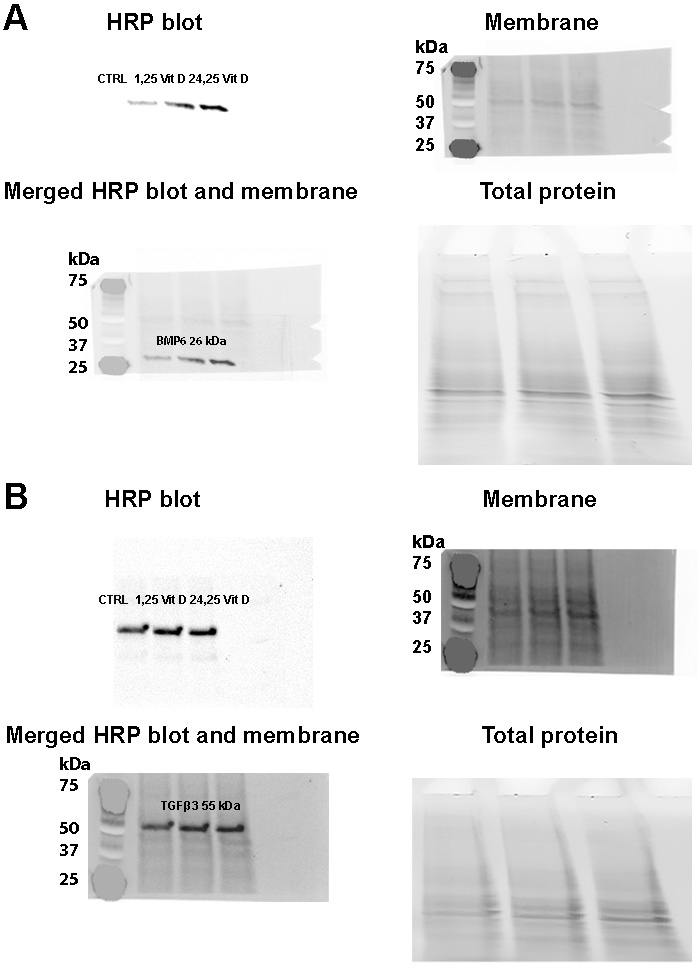

Supplement: Supplementary file 1 [file cells-14-01583-s001.zip › Figure S4.jpg]
